# Dual Effects of Hypoxia-Inducible Factors-1 Alpha in Bleomycin-Induced Pulmonary Fibrosis Treated by Human Umbilical Cord Mesenchymal Stem Cells

**DOI:** 10.1155/2021/6658855

**Published:** 2021-11-26

**Authors:** Hongpeng Zhang, Hao Wang, Yong Xia, Nianmin Qi

**Affiliations:** Asia Stem Cell Regenerative Pharmaceutical Co. Ltd., 2066 Wangyuan Road, Shanghai 201499, China

## Abstract

Pulmonary fibrosis (PF) is a kind of lung disease characterized by scar formation and inflammation damage. Mesenchymal stem cells (MSCs) are considered a promising therapy because of multidirectional differentiation and immune regulation. Our research was designed for identifying the preventative defensive ability and therapeutic effect of human umbilical cord mesenchymal stem cells (HUCMSCs). HUCMSCs were administered before or after bleomycin injection in different groups of C57BL/6 mice. We calculated the survival time of mice, the lung coefficients, contents of hydroxyproline, and pathological scores. The expression levels of HIF-1*α* (hypoxia-inducible factor-1*α*), *α*-SMA (*α*-smooth muscle actin), *γ*H_2_AFX (*γ*H2A histone family, member X), ZO-1 (zonula occludens-1), ROS (reactive oxygen species) content, and proliferation ability of A549 cells were detected after treatment with bleomycin and HUCMSCs conditioned medium (HUCMSCs-CM), respectively, or together *in vitro*. In addition, we examined the secretome of HUCMSCs in regular and inflammatory stimulation conditions. Our results demonstrated that prophylactic HUCMSC administration before bleomycin-induced modeling process could significantly meliorate damage to pulmonary fibrosis. After the deletion of HIF-1*α*, damage markers in A549 cells were significantly reduced in therapeutic administration condition. However, it was the opposite in prophylactic administration condition. The results confirmed that HUCMSCs had available preventive effect on bleomycin-induced pulmonary fibrosis *in vivo* and *in vitro*. However, it may have a negative effect in therapeutic administration condition because of the dual effect of HIF-1*α*.

## 1. Introduction

Pulmonary fibrosis (PF) is a large class of lung diseases characterized by excessive inflammation, fibroblast proliferation, and aggregation of extracellular matrix (ECM) [1]. Current treatment of pulmonary fibrosis is highly controversial. It is very difficult to cure when serious fibrosis symptoms have appeared [2]. Therefore, searching a new way to prevent the formation of pulmonary fibrosis rather than repression is actually a better way.

Mesenchymal stem cells (MSCs) are a group of multifunctional stem cells which include self-replication, multidirectional differentiation potential, and immune regulation function. Currently, autologous bone marrow mesenchymal stem cells (BMSCs) and adipose-derived mesenchymal stem cells (ADSCs) have been widely deemed to potential medicines [3]. Besides the advantages of BMSCs, umbilical cord mesenchymal stem cells (UCMSCs) have many other characteristics such as more widespread source, more stable effect regarding medicines, and better modification potential [4]. Therefore, using UCMSCs for prophylactic treatment in the inflammatory period before fibrosis symptoms may be a more effective way for the treatment of PF in the future. It is of great significance for the treatment of pulmonary fibrosis clinically.

Hypoxia-inducible factor-1 (HIF-1) plays a central role in the stimulation of hypoxic condition. Stable HIF-1 dimerizes were translocated to the nucleus, for inducing the activation of the gene containing the hyperoxia-responsive elements in promoters [5]. However, it is very interesting that HIF-1 has dual effect characteristics probably. On the one hand, many researches have proved that HIF-1 can participate in the abnormal fibrosis pathway stimulated by TGF-*β*1 [6]. On the other hand, the expression of HIF-1 can also inhibit the injury of fibroblasts and epithelial cells, prolong cell survival time, and inhibit apoptosis [7]. In addition, HIF-1 has also been confirmed to promote the survival, migration, and differentiation of MSCs, thereby playing a therapeutic role. All these show that HIF-1 may play two different roles in pulmonary fibrosis condition.

The aim at the present study was to confirm that HUCMSCs have preventive or therapeutic effects on bleomycin-induced pulmonary fibrosis *in vivo* and *in vitro*, and HIF-1*α* played a key role in this process.

## 2. Materials and Methods

### 2.1. Culture and Purification of HUCMSCs

The study was approved by the ethical committee of the Shanghai Jiao Tong University (Shanghai, China). HUCMSCs were purified from umbilical cord of newborn infant donors and cultured in our laboratory. Briefly, a fresh umbilical cord was harvested from newborn healthy infant; the umbilical cord was cubed into 1.0-2.0 mm^3^ pieces and then digested with 0.1% Collagenase I (Thermo Fisher) and 0.2% Hyaluronidase (Thermo Fisher); HUCMSCs are resuspended in*α*-MEM culture medium (Gibco) with 10% fetal bovine serum (FBS) (Gibco). Finally, cell was seeded at a density of 1.5 × 10^5^ cells per T175 Flasks. Cells were cultured in a humidified atmosphere of air (95%) and CO_2_ (5%) at 37°C.

### 2.2. Cell Immunophenotype Identification

Collected Passage 2 HUCMSCs by digesting cell with 0.25% trypLE Express (Gibco), incubated with R-phycoerythrin- (PE-) conjugated antibodies against human CD73, CD90, CD105, CD14, CD19, CD34, CD45, and HLA-DR (each 1 *μ*g/100 *μ*l, CST) for 30 min on ice. Flow Cytometer (cytoFLEX, BeckmanCoulter) was applied to verify the surface antigen protein.

### 2.3. Ostogenic Induction

The HUCMSCs were seeded in six-well plates at a density of 1 × 10^5^ cells/well. The growth medium supplemented with 10 mmol/l sodium *β*-glycerophosphate, 1 *μ*mol/l dexamethasone, and 50 *μ*mol/l vitamin C was used as an osteogenic medium. Cells were cultured in osteogenic medium for 4 weeks. The cultures were stained with Alizarin Red S (Sigma) to detect mineralized nodule.

### 2.4. Adipogenic Induction

The growth medium supplemented with 0.5 mmol/l isobutyl-methylxanthine, 200 *μ*mol/l indomethacin, 1 *μ*mol/l dexamethasone, and 10 *μ*mol/l insulin was used as an adipogenic medium. Cells were cultured in an adipogenic medium for 1 week. The cultures were stained with Oil Red O (Sigma) to detect lipid droplets.

### 2.5. Chondrogenic Induction

The growth medium supplemented with 10 *μ*g/l transforming growth factor-*β*1, 0.1 *μ*mol/l dexamethasone, 100 *μ*mol/l ascorbic acids, 200 mmol/l L-glutamine, and 1% ITS+Premix (Gibco) was used as a chondrogenic medium. Cells were cultured in a chondrogenic medium for 3 weeks. The cultures were stained with Alcian Blue (Sigma) to detect cartilage tissue.

#### 2.5.1. Mouse Pulmonary Fibrosis Model Preparation

Since bleomycin has been regarded as the most classical modeling method of PF, we decided to use it in our experiment. Briefly, the C57BL/6 female mice weighting 18–20 g used in this study were obtained from the Experimental Animal Center of the Shanghai Jiao Tong University. Mice were anesthetized by transperitoneal injection of 1.5% pentobarbital sodium. The skin was split, the neck tissue was separated to expose the trachea, and then, 0.75 U/kg bleomycin was injected into the trachea. The body position of mice was switched repeatedly to distribute evenly the bleomycin in the trachea. The sham group was conducted in the same operation with the injecting of PBS. The control group had not been operated. All animal experiments were conducted in accordance with the committee guidelines of the Shanghai Jiao Tong University for animal experiments.

### 2.6. HUCMSC Administration Schedule

Mice were randomly divided into 5 groups (12 animals in each group, female, 18-20 g weight): control (untreated), sham, model (injected 0.75 U/kg bleomycin into the trachea for pulmonary fibrosis), pretreatment with HUCMSCs (preventative therapy with HUCMSCs before bleomycin injection), and treatment with HUCMSCs (therapy with HUCMSCs after bleomycin injection). 1 × 10^7^ cells/kg HUCMSCs (0.3 ml) were administrated to every mouse in the group pretreated with HUCMSCs by tail vain injection in the date of 7 days before operation and 1 hour and 7 days after operation. 1 × 10^7^ cells/kg HUCMSCs (0.3 ml) were administrated to every mouse in the group treated with HUCMSCs by tail vain injection in the date of 1 hour and 7 and 14 days after operation. The day started operation was regarded as Day 1. The survival time of mice daily (in total 21 days) was observed and recorded. If the mouse died unexpectedly, we acquired the blood and lung tissue of the dead mouse in advance.

### 2.7. Histological Analysis

All mice were executed after 21 days. We acquired the blood and lung tissue and counted the lung coefficient (ratio of wet lung weight to body weight). The right lung of all mice was infused with of 4% neutral buffered formalin solution. The fixed lungs were embedded in paraffin, sectioned at a thickness of 5 *μ*m, and stained with hematoxylin and eosin (HE) and Masson's trichrome. A random number was assigned to every lung section from the five groups. An experienced pathologist blinded to the random numbers evaluated the slides for the degree of pulmonary fibrosis.

### 2.8. Pulmonary Fibrosis Index Analysis

The left lungs of mice were applied with hydroxyproline and TNF-*α* assay; the operation was carried out as instructed (Shanghai Haling Biological Technology Co, Ltd.).

### 2.9. A549 Cell Culture and Treatment

Cell lines of human lung adenocarcinoma epithelial cells A549 cells were cultured in DMEM with 10% fetal bovine serum (Gibco) and 1% antibiotics (100 U/ml penicillin, 0.1 mg/ml streptomycin). The HUCMSC conditional medium (CM-HUCMSCs) was the supernatant of the HUCMSC medium that has been used to culture HUCMSCs for 24 hours. In the control group, A549 cells were culture commonly. In the CM-pretreatment group and CM-treatment group, A549 cells were cultured with HUCMSCs-CM for 48 hours. In the bleocymin group, the A549 cells were treated with bleomycin at the final concentration of 12 *μ*g/ml for 6 h. In the bleocymin+CM-pretreatment group, A549 cells were cultured with HUCMSCs-CM for 48 hours then replaced with DMEM containing bleomycin with final concentration of 12 *μ*g/ml for 6 hours. In the bleocymin+CM-treatment group, the A549 cells were treated with bleomycin at the final concentration of 12 *μ*g/ml for 6 hours then cultured with HUCMSCs-CM for 48 hours. siRNA was used to inhibit the function of HIF-1*α* gene.

### 2.10. Western Blot

Total protein from A549 cells were collected and extracted with RIPA. The protein concentration was quantified by BCA protein quantitative. The total protein was separated on SDS-PAGE gel of 10% (absin, abs9324) or 15% (absin, abs9325) and transferred to PVDF membrane. It is blocked with 5% BSA for 2 h at room temperature and incubated with primary antibodies at 4°C for overnight against HIF-1*α* (Bioss, bsm-51085 M), *α*-SMA (elabscience, E-AB-34268), ZO-1 (FineTest, FNab09750), *γ*H_2_AFX (Millipore, 05–636), and GAPDH (EnoGene, E12-052-3). HRP-conjugated second antibodies were incubated at room temperature for 2 hours. Proteins were visualized with ECL detection reagents after washing in PBST.

### 2.11. Real-Time Quantitative PCR

Total RNA extracted from cells with TRIzol. The total RNA concentration was detected by ultraviolet spectrophotometer. Reverse transcription was completed with First-Strand cDNA Synthesis Kit (Biomiga, RT0212-01) according to the manufacturer's protocol. Real-time quantitative PCR was completed with SYBR Green qPCR Mix (Beyotime, D7265) in ABI StepOne Detection System. The primer sequences are listed in Table 1. All of the samples were read in triplicate, and data were normalized to GAPDH.

### 2.12. Immunofluorescence Analysis

A549 cells were cultured in 6-well plates and treated with bleomycin. These cells after crawled on the slide were used to fix for 30 min with 4% paraformaldehyde. 0.5% Triton X-100 was used to break the membrane for 30 min. The cells were blocked in 5%BSA for 1 h and then incubated with primary antibody at 4°C overnight against TP53BP1 (Boster, BA2878). The slides were washed with PBST and incubated with second antibody for 2 h at room temperature. Cells were stained with DAPI for 5 min. Immunofluorescence laser microscopy was use to observed these cells after washing in PBST.

#### 2.12.1. ROS Detection by 2′-7′-Dichlorofluorescein Diacetate (DA-DCFH)

2 × 10^7^ well A549 cells were cultured in 6-well plates in serum-free medium (Sino Biological Inc., M293TIS-1). DCFH-DA reagent (Jiancheng biology, Nanjing, China) was diluted with serum-free medium in the ratio of 1 : 1000 to the final concentration of 10 *μ*m. 1 ml of serum-free medium containing DCFH-DA was added into 6-well plate and incubated at 37°C for 1 h. The plates were washed with PBS for three times. They were excited with a 500 nm wavelength laser, and the fluorescence emission was detected at 525 nm.

### 2.13. Cell Viability Assays

Cell viability was determined with Cell Counting Kit-8 reagents (M4839, AbMole). A549 cells were divided into the control, CM-pretreatment, CM-treatment, bleomycin, bleomycin+CM-pretreatment, and bleomycin+CM-treatment groups just like the previous experiments. siRNA was used to inhibit the function of HIF-1*α*. A549 cells (5 × 10^3^ cells/well) were seeded in 96 well plates after treatment. The culture volume of each well was 100 *μ*l. 10 *μ*l CCK-8 reagent was added and incubated after treatment at 37°C for 2 hours after 5 days. The OD450 of each well was measured with a Multimode Reader.

### 2.14. Secretome of HUCMSC Analysis

The HUCMSC conditional medium (CM-HUCMSCs) were the supernatant of the HUCMSC medium that have been used to culture HUCMSCs for 24 hours regularly. The induced HUCMSC conditioned medium (ICM-HUCMSCs) were the supernatant of the HUCMSC medium that have been used to culture HUCMSCs for 24 hours with 1000 IU/ml IFN-*γ* and 500 IU/ml TNF-*α*. ELISA were used to detect IL-6, IL-10, HGF (hepatocyte growth factor), IDO-1 (indoleamine2,3-dioxygenase-1), TGF-*β*1 (transforming growth factor-*β*), VEGF (vascular endothelial growth factor), PGE-2 (Prostaglandin E2), and CCL-2 in both CM-HUCMSCs and ICM-HUCMSCs.

### 2.15. Data Analysis

Statistical analysis was performed with SPSS 22. Multigroup comparisons were analyzed using one-way ANOVA. Independent data for 2 groups were analyzed by *t*-test. *P* value < 0.05 was considered statistically significant. *p* value < 0.01 was considered statistically significant. The figures were performed by GraphPad Prism 7.

## 3. Results

### 3.1. Isolation, Purification, and Identification of HUCMSCs

**Figure 1:**
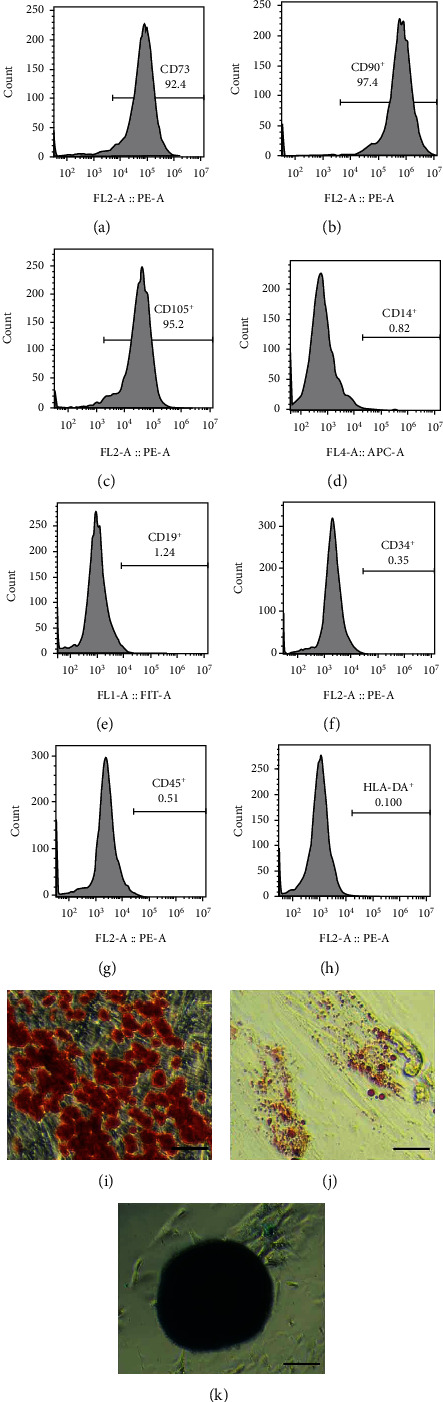
Detection of surface markers, osteogenesis, adipogenesis, and cartilage-induced differentiation of HUCMSCs. FACS was used to detect the surface markers, and ICC was used to detect the differentiation. (a–c) The expression of positive surface marker CD73, CD90, and CD105. (d–h) The expression of surface negative markers CD14, CD19, CD34, CD45, and HLA-DR. (i) The osteogenic differentiation was analyzed by Alizarin Red staining. (j) The adipogenic differentiation was analyzed by Oil Red O staining. (j) The chondrogenic differentiation was analyzed by Alcian Blue staining. The scale bar = 20 *μ*m in (i, j) and 100 *μ*m in (k).

HUCMSC surface markers were detected by FACS, adipogenic differentiation was analyzed by Oil Red O staining, osteogenic differentiation was analyzed by Alizarin Red staining, and chondrogenic differentiation was analyzed by Alcian Blue staining (Figures 1(i)–1(k)). The HUCMSCs were positive for mesenchymal surface markers CD73, CD90, and CD105 (Figure 1(a)–1(c)), negative for markers CD14, CD19, CD34, CD45, HLA-DR (Figures 1(d)–1(h)) and had multidifferentiation potentials toward osteoblasts, adipocytes, and chondroblasts. The results showed that the HUCMSCs have high purity and excellent differentiation potential.

### 3.2. Evaluation of Bleomycin-Induced Pulmonary Fibrosis in Mice

**Figure 2:**
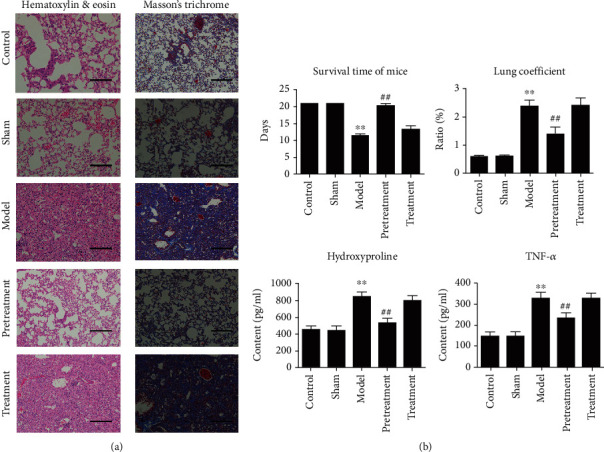
Analysis of several indexes in the evaluation of HUCMSC administration of bleomycin-induced pulmonary fibrosis in mice. (a) Results of HE and Masson staining in mouse lungs of the control, sham, model, pretreatment, and treatment groups. Scale bar = 100 *μ*m. (b) Results in the control, sham, model, pretreatment, and treatment groups. Data are presented as mean ± SEM. ^∗^*p* ≤ 0.05, ^∗∗^*p* ≤ 0.01 vs. the control; ^#^*p* ≤ 0.05, ^##^*p* ≤ 0.01 vs. the model.

The average survival time of bleomycin-induced model group was 11.5 days, which was very significantly lower than that of the control group (21 days) (Figure 2(b)). The average lung coefficient of the model group was 2.39%, which was very significantly higher than that of the control group (0.59%) (Figure 2(b)). The average content of hydroxyproline was 840 pg/ml in the model group, which was very significantly higher than 446 pg/ml in the control group (Figure 2(b)). The results of HE and Masson's trichrome staining showed that the alveolar wall of the control group mice was intact and the alveolar septum was normal (Figure 2(a)). In the model group, tissue edema, wide spacing of bubbles, and a large amount of deposition of extracellular matrix were observed (Figure 2(a)). From the above data, it can be proven that bleomycin-induced mouse model is successful and can reflect the characteristics of pulmonary fibrosis.

### 3.3. Administrated HUCMSCs before Bleomycin-Induced Operation Effectively Alleviated Pulmonary Fibrosis Symptom in Mice

Two HUCMSC administration schedules were tested in our research: prophylactic administration before bleomycin injection (pretreatment group) and therapeutic administration after it (treatment group). The results showed the average survival time of mice in pretreatment with the HUCMSC group was 20.3 days, which was very significantly higher than that in the model group (11.5 days). The average survival time of mice in the treatment with the HUCMSC group was 13.3 days, which was not significantly different from that in the model group (Figure 2(b)). The average lung coefficient of the pretreatment group was 1.4%, which was very significantly lower than that of the model group (2.39%) (Figure 2(b)). The lung coefficient of the treatment group was 2.42%, which was not significantly different from that of the model group. The average content of hydroxyproline in the pretreatment group was 526 pg/ml, which was very significantly lower than that in the model group (840 pg/ml) and closer to that in the control group (446 pg/ml). The content of hydroxyproline in the treatment group was 787 pg/ml, which was not significantly different from that in the model group (840 pg/ml) (Figure 2(b)). The results show that prophylactic administration (pretreatment group) can alleviate the injury of bleomycin-induced pulmonary fibrosis, but it is not effective to treat pulmonary fibrosis after successful modeling.

### 3.4. Effects of HUCMSCs-CM and Bleomycin in A549 Cells

**Figure 3:**
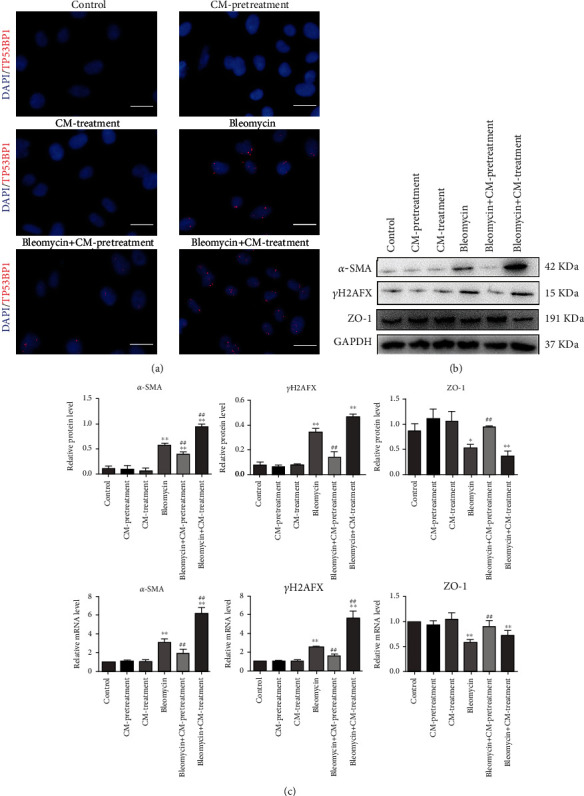
Preventive administration of HUCMSCs can effectively attenuate pulmonary fibrosis induced by bleomycin in A549 cells. (a) Results of immunofluorescence staining in A549 cells of the control, CM-pretreatment, CM-treatment, belomycin, belomycin+CM-pretreatment, and belomycin+CM-treatment groups. TP53BP1 are stained in A549 cells with antibodies (red). Nuclei are stained with DAPI (blue). Scale bar = 100 *μ*m. (b) Results of western blot in the same groups. (c) Results of qPCR in the same groups. Data are presented as mean ± SEM. ^∗^*p* ≤ 0.05, ^∗∗^*p* ≤ 0.01 vs. the control; ^#^*p* ≤ 0.05, ^##^*p* ≤ 0.01 vs. the model.

Two HUCMSC administration schedules *in vitro* were tested in our research: prophylactic administration before bleomycin injection (pretreatment group) and therapeutic administration after it (treatment group). TP53BP1 is a reliable marker for double-strand breaks (DSBs) in cells. The expression of TP53BP1 in the cell nucleus of the pretreatment group decreased compared with the control group (Figure 3(a)). However, the expression of TP53BP1 in the cell nucleus of the treatment group increased compared with the control group (Figure 3(a)). The results showed that HUCMSCs-CM preventive administration can prevent bleomycin-induced DSBs effectively. Additionally, HUCMSCs-CM therapeutic treatment may aggravate DSBs, at least did not attenuate it.

qPCR and WB were used in detecting the expression of *α*-SMA, *γ*H_2_AFX, and ZO-1. There were no significant differences in the expression of *α*-SMA, *γ*H_2_AFX, and ZO-1 in the condition that only treated with HUCMSCs-CM for 48 h compared with the control group, either prophylactically or therapeutically (Figures 3(b) and 3(c)). When cells were only treated with 12 *μ*g/ml bleomycin for 6 h, *α*-SMA and *γ*H_2_AFX were significantly upregulated compared with the control group and ZO-1 was downregulated. There were no significant differences in the expression of *α*-SMA, *γ*H_2_AFX, and ZO-1 between the pretreatment group and control group. Meanwhile, the expression of *α*-SMA, *γ*H_2_AFX, and ZO-1 in the mRNA and protein levels was very significantly different in control group and treatment group. The data showed HUCMSCs-CM has a preventative defensive effect on bleomycin-induced pulmonary fibrosis in A549 cells, which can effectively prevent ECM accumulation, EMT, and DSBs. However, there were no obvious therapeutic effects on the cells with pulmonary fibrosis.

### 3.5. Function of HIF-1*α* Protein in Stimulation of HUCMSCs-CM and Bleomycin in A549 Cells

**Figure 4:**
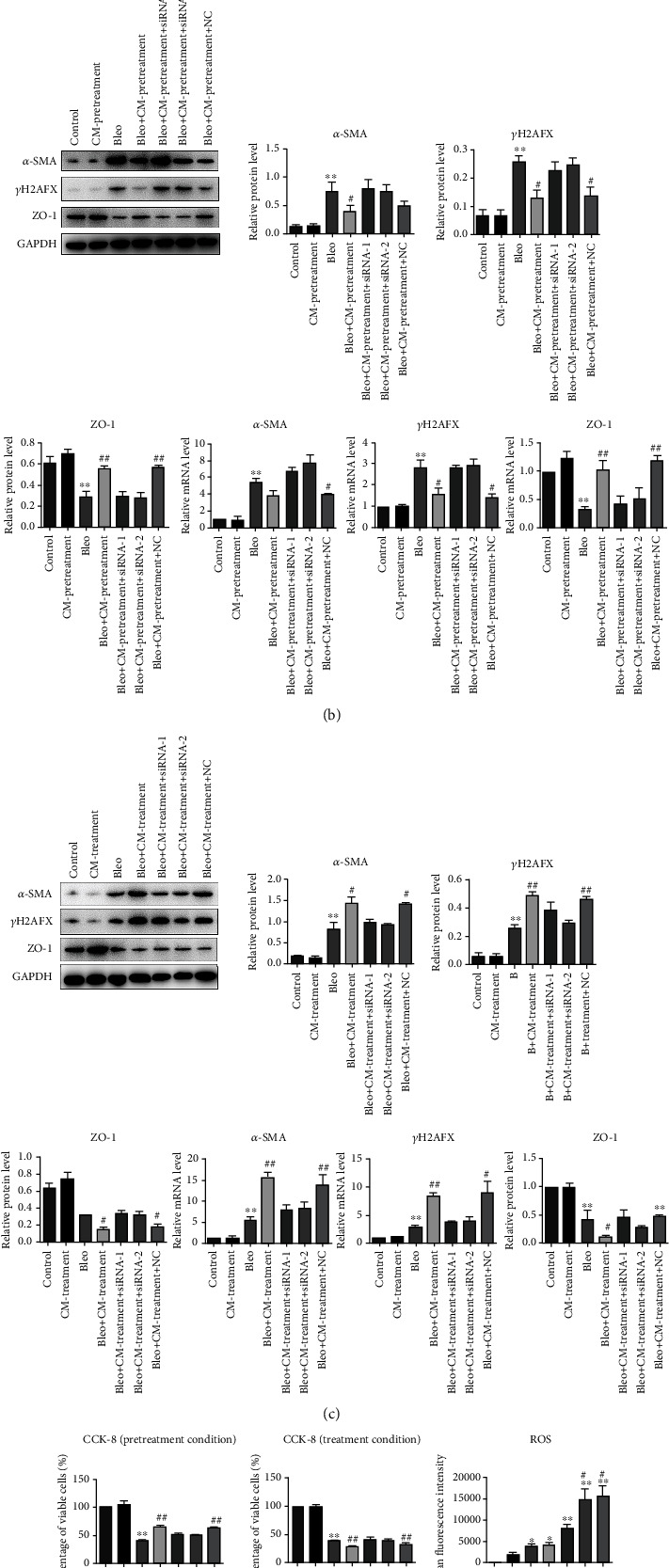
Analysis of several indexes in the evaluation of HUCMSC administration of bleomycin-induced pulmonary fibrosis in A549 cells. (a) HIF-1*α* results and analysis of western blot and qPCR in A549 cells. (b) Cellular marker *γ*H2AFX, ZO-1, and *α*-SMA results and analysis of western blot and qPCR in the pretreatment condition. (c) Cellular marker *γ*H2AFX, ZO-1, and *α*-SMA results and analysis of western blot and qPCR in treatment condition. (d) Cell viability assays and ROS results and analysis in the pretreatment and treatment conditions. Data are presented as mean ± SEM. ^∗^*p* ≤ 0.05, ^∗∗^*p* ≤ 0.01 vs. the control; ^#^*p* ≤ 0.05, ^##^*p* ≤ 0.01 vs. the model.

The expression of HIF-1*α* in A549 cells was detected in qPCR and WB. Unlike qPCR level, HIF-1*α* had a low expression in WB (Figure 4(a)). Under the induction of bleomycin, protein expression levels of HIF-1*α* were significantly upregulated, but mRNA expression level was not (Figure 4(a)). No matter prophylactically or therapeutically, the protein expression level of HIF-1*α* increased significantly when cells were only treated with HUCMSCs-CM (Figure 4(a)). Meanwhile, the expression level of HIF-1*α* in the pretreatment group and treatment group was significantly higher than that in the model group and control group (Figure 4(a)). The results indicated that HIF-1*α* may play an important role in the stimulation of HUCMSC-CM for belomycin-induced pulmonary fibrosis.

DA-DCFH reagent and fluorescence ELISA were used to detect ROS content in A549 cells *in vitro*. The contents in the CM-pretreatment, CM-treatment, and model groups were significantly higher than that in the control group (Figure 4(d)). Also, in the pretreatment group and the treatment group, the contents of ROS were significantly higher than that in the control group and the model group (Figure 4(d)). The results confirmed that the content of ROS in A549 cells were consistent with the expression level of HIF-1*α*, which indicated that ROS probably play an important role in the stable existence of HIF-1*α*.

### 3.6. HUCMSCs-CM Regulated Bleomycin-Induced Pulmonary Fibrosis via HIF-1*α*

We observed the changes of markers after knocking down HIF-1*α* mRNA and inhibiting function. The expression level of *α*-SMA and *γ*H_2_AFX in the treatment group was significantly decreased by knocking down the expression level of HIF-1*α* in WB and qPCR compared with the control group (Figure 4(c)). Additionally, it can significantly upregulate the expression level of ZO-1 (Figure 4(c)). By contrast, the expression level of *α*-SMA and *γ*H_2_AFX in the pretreatment group was significantly increased compared with the control group (Figure 4(b)). Meanwhile, ZO-1 in the pretreatment group was significantly decreased (Figure 4(b)). The results suggested that HIF-1*α* played a key role in the process of HUCMSCs-CM alleviating fibrosis symptoms. But the effects may be completely different in the process of preventive and therapeutic condition.

Similar effects can also be observed in cell viability assays. There was no significant difference in cell viability between the control group and the CM-pretreatment/CM-treatment group after common culture of 5 days (Figure 4(d)). The viability rate of the belomycin group was significantly lower than the control group (Figure 4(d)). Meanwhile, in the condition of prophylactic administration of bleomycin and HUCMSCs-CM, the cell viability rate was significantly higher than that of the belomycin group, but the viability rate of the belomycin+CM-treatment group was significantly lower than that of the bleomycin group (Figure 4(d)). These two different trends will be ameliorated by siRNA.

### 3.7. Secretome of HUCMSCs and Treatment of Pulmonary Fibrosis

**Figure 5:**
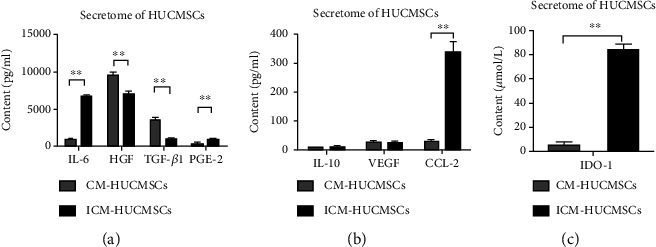
Analysis of several factors in the secretome of HUCMSCs. (a) ELISA results of IL-6, HGF, TGF-*β*1, and PGE-2 in CM-HUCMSCs and ICM-HUCMSCs. (b) ELISA results of IL-10, VEGF, and CCL-2 in CM-HUCMSCs and ICM-HUCMSCs. (c) ELISA results of IDO-1 in CM-HUCMSCs and ICM-HUCMSCs. Data are presented as mean ± SEM. ^∗^*p* ≤ 0.05, ^∗∗^*p* ≤ 0.01.

In order to further investigate dual effects of HUCMSCs via HIF-1*α* in the bleomycin-induced pulmonary fibrosis, we detected the expression levels of factors in the secretome of HUCMSCs in inflammatory stimulation condition (ICM-HUCMSCs) and common condition (CM-HUCMSCs) (Figure 5). There was no significant difference in IL-10 and VEGF between CM-HUCMSCs and ICM-HUCMSCs (Figure 5(b)). The secretion of IL-6, PGE-2, IDO-1, and CCL-2 were very significantly upregulated in the inflammatory environment (Figure 5). Meanwhile, the secretion of HGF and TGF-*β*1 was significantly downregulated after inflammatory factor stimulation (Figure 5(a)).

## 4. Discussion

In the current study, our research demonstrated the effects of HUCMSCs and HUCMSCs-CM on bleomycin-induced pulmonary fibrosis *in vivo* and *in vitro*. The results *in vivo* showed that prophylactic administration of HUCMSCs before the modeling process could significantly reduce the symptoms of pulmonary fibrosis and the accumulation of ECM. However, HUCMSCs did not show a significant therapeutic effect. The results *in vitro* suggested that HIF-1*α* had dual effects (positive and negative) under the different administration conditions of bleomycin and HUCMSCs-CM.

Bleomycin induction is the most popular used method of pulmonary fibrosis modeling among the many fibrotic agents/drugs such as radiation, silica, asbestos, and transgenic model animals [8]. Bleomycin is a robust chemotherapeutic antibiotic that can disrupt the cellular cycle by cleaving single- and double-stranded DNA [9]. The PF models induced with it demonstrate high reproducibility, accessibility, and histological similarity to the real disease. A549 cells retain the features of type II alveolar epithelial cells despite belonging to cancer cells [10]. Therefore, the mechanism of pathogenesis of pulmonary fibrosis was investigated in bleomycin-induced A549 cells. In our results, the expression changes of cellular senescence marker *γ*H2AFX, cell damage marker TP53BP1, and cell fibrosis marker *α*-SMA were consistent with the former studies of bleomycin on A549 cells [11]. Our results verified the protein expression of HIF-1*α* had a positive correlation with the content of ROS in A549 cells. These results recognized the previous experiment that ROS can effectively inhibited the activity of prolyl hydroxylases (PhD) and improved the stability of HIF-1*α* peptides [12].

Some studies showed MSCs can treat pulmonary fibrosis, but the outcomes were not always desirable. Our results verified that HIF-1*α* plays different effects in different periods of pulmonary fibrosis, which may be one of the reasons. On the one hand, previous studies have suggested that HIF-1*α* can induce collagen I, reduce the expression of matrix metalloproteinase-2 (MMP2), and increase plasminogen activator inhibitor-1 (PAI-1), tissue metalloproteinase-1 inhibitor (TIMP-1), and connective tissue growth factor (CTGF) [13]. On the other hand, HIF-1*α* in the abnormal inflammatory environment also can protect fibroblasts and epithelial cells [7]. Our results confirmed that HIF-1*α* improved the expression of marker of pulmonary fibrosis in bleomycin-induced condition *in vitro*. However, HIF-1*α* also had inhibitory effects on the fibrotic marker in the condition of HUCMSCs-CM prophylactic administration. This conclusion suggested that HIF-1*α* may play an anti-inflammatory function in preventive conditions which can inhibit a fibrotic marker effectively. The results of cell viability assay *in vitro* also illustrated this viewpoint. The results are consistent with the downregulation of TNF-*α* in the prevention group *in vivo*.

In our research, we treated the PF model with completely different administration methods in the preventive administration group and the therapeutic administration group. The dosage and time frame design of these two methods mainly refer to the former researches [14, 15] and our own previous results. The results of previous studies showed that the bleomycin-induced PF model reached its peak after 7 days, transformed from inflammation to fibrosis after 10 days, and reached the peak of fibrosis on the 14th day. Therefore, we decided that the administration timeframe in the treatment group was designed on day 0, day 7, and day 14. According to the Phinney et al., early administration is conducive to the treatment of PF [16]. Therefore, we decided that the administration timeframe in the pretreatment group was designed on day -7, day 0, and day 7.

We tested the secretome of HUCMSCs to explore the pathway that determines HIF-1*α* plays dual effects. Some factors were determined in the detection of secretome through published papers. The results showed that the secretome of HUCMSC change significantly in the inflammatory stimulation condition compared with the regular condition. Pan et al. claimed MSCs have a short survival time *in vivo*, and more than 90% of the cells will be removed in 3 days of administration [17]. Secretome of HUCMSCs of preventative defensive administration may be in a noninflammatory common condition, and HGF and TGF-*β*1 in the secretome play major work. Many studies suggested that HIF-1*α* may promote expression levels of HGF and TGF-*β*1 in bleomycin-induced condition, which are consistent with our data [18, 19]. However, the secretome of HUCMSCs of therapeutic administration may be affected by the abnormal inflammatory enviroment, so IL-6, PGE-2, IDO-1, and CCL-2 in the secretome may play major work. Ren et al. considered HIF-1*α* may upregulate the expression of CCL2 and promote the inflammation and fibrosis symptoms induced by bleomycin, which was also consistent with our result [20].

In conclusion, our results confirmed that HUCMSCs and HUCMSCs-CM had available defensive effect on bleomycin-induced pulmonary fibrosis in mice. It is of great significance for the treatment of pulmonary fibrosis clinically.

## Figures and Tables

**Table 1:** Primer and siRNA sequences.

Nucleotide name	Forward (5′-3′)	Reverse (5′-3′)
HIF-1*α*	GTCTGAGGGGACAGGAGGAT	CTCCTCAGGTGGCTTGTCAG
*α*-SMA	CCGACCGAATGCAGAAGGA	ACAGAGTATTTGCGCTCCGAA
*γ*H2AFX	TGGCTATGTGGACAGCAAGAGTCGTTT	GGTGCTTGGATTGCCGAGTTGAGT
ZO-1	CATCTCCAGTCCCTTACCTTTCG	TGGTTCTGCCTCATCATTTCCTC
GAPDH	ACAGTCCATGCCATCACTGCC	CCATCCAATCGGTAGTAGCC
HIF-1*α* siRNA-1	GAAGGUCUAGGAAACUCAAdTdT	UUGAGUUUCCUAGACCUUCdTdT
HIF-1*α* siRNA-2	GAAGGUCUAGGAAAACUCAAdTdT	UUGAGUUUCCUAGACCUUCdTdT

## Data Availability

The data used to support the findings of this study are included within the article.
